# Evaluating the relationship between lactate levels during coronary artery bypass graft surgery and postoperative renal dysfunction

**DOI:** 10.34172/jcvtr.33051

**Published:** 2024-06-25

**Authors:** Fatemehshima Hadipourzadeh, Roxana Rastravan, Ziae Totonchi, Evaz Heydarpur, Zahra Faritous

**Affiliations:** Rajaie Cardiovascular Medical and Research Institute, Iran University of Medical Sciences, Tehran, Iran

**Keywords:** Lactate, Cardiac surgery, Renal dysfunction

## Abstract

**Introduction:**

Postoperative Acute renal failure related to cardiac surgery is a common complication due to cardiac surgery and is estimated to influence up to 30% of patients. Serum lactate is a famous biomarker of tissue ischemia and is regularly checked during surgery.

**Methods:**

In this retrospective observational research, the records of 395 patients undergoing CABG were examined. Patients were classified into 4 groups based on the difference between the maximum lactate level measured during surgery and its baseline level. Also creatinine and urea levels, blood sugar, hemoglobin, and hematocrit pre, postoperative were recorded. The intraoperative and postoperative use of inotropes and the durations of surgery, cardiopulmonary bypass pump, and aortic cross-clamping were also recorded.

**Results:**

According to the results, pre, post and 24-hour postoperative blood urea nitrogen were not significantly related to intraoperative lactate changes. Also, pre and 24-hour postoperative creatinine had no significant relationship with intraoperative lactate changes, while postoperative creatinine was significantly associated with intraoperative lactate changes (*P* value=0.05). The duration of cardiopulmonary bypass (*P* value=0.02), intraoperative inotrope infusion (*P* value=0.03), inotrope infusion during the first six hours in ICU (*P* value=0.049), and receiving packed cell (*P* value=0.006) and receiving platelets during surgery (*P* value=0.04) were significantly related to intraoperative lactate changes. Furthermore, no significant relationship was observed between the duration of hospitalization in the ICU and the hospital and intraoperative lactate changes.

**Conclusion:**

According to the results, blood lactate level is an unreliable marker for predicting renal dysfunction postoperative.

## Introduction

 Acute renal failure (ARF) is among the most serious and potentially lethal complications after cardiac surgery and its early diagnosis leads to quick treatment and prevention of renal damage progression. Despite preventive and therapeutic strategies, it is extremely important to detect patients at risk. Acute renal failure after cardiac surgery can be attributed to reduced tissue perfusion, ischemia, and the negative effects of free radicals and angiotensin that stimulate vasoconstriction, such as endothelins.^1,2^

 The risk factors for acute renal failure are multifactorial, such as old age, chronic renal diseases, diabetes, long duration of surgery, and long duration of cardiopulmonary bypass. The pathophysiology of acute renal failure after cardiac surgery is complicated, and the mechanism of renal damage include micro embolization, neuro hormonal activation, exogenous and endogenous toxins, metabolic, hemodynamic, and inflammatory factors, ischemic-reperfusion injury, and oxidative stress. These injury mechanisms can be interconnected and have a synergistic effect.^3,4^

 The prevalence of acute renal failure after cardiac surgery has been reported to be 30%, independently leading to enhanced mortality and morbidity. Even as light increase in serum creatinine after cardiac surgery is independently associated with by enhanced postoperative mortality. Cardiac surgery, similar to any other surgery impacts other body organs.^1,5^

 The relationship between lactate biomarkers and patients with acute renal injury after cardiac surgery has been proven. Serum lactate, as a well-known biomarker of tissue ischemia, is regularly checked during surgery. Previous studies have assessed the serum lactate level and have demonstrated a relationship between the levels of this biomarker and patients with acute renal injury after cardiacsurgery.^6^

 The normal blood lactate level is 0-2 mmol/L, and hyperlactatemia is generally defined as a value higher than 3.5 mmol/L.^7^ Due to their unique blood circulation, kidneys are susceptible to ischemic injury, in which the renal medulla is typically perfused at a low oxygen pressure with limited reserve.^8^ Considerable increases in serum lactate may stem from renal tissues due to hypoperfusion during cardiac revascularization surgery.^6^ In patients undergoing cardiac surgery, postoperative lactate levels are associated with higher mortality, prolonged hospitalization in the intensive care unit (ICU) and the hospital, more hours of mechanical ventilation, the need for higher-dose inotrope, and the need for alternative measures of renalfunction.^9^ Several factors contribute to postoperative lactate production and metabolism, stemming from hypoxic and non-hypoxic mechanisms; it is generally believed that the clinical effect of heightened lactate levels resulting from tissue hypoxia is greater than that resulting from non-hypoxic causes.^7^

 The present study aims to assess the relationship between intraoperative serum lactate levels and renal function after coronary artery bypass surgery.

## Materials and Method

###  Study type, population, and data collection

 This research was conducted as an observational retrospective study after approval from the ethics committee (code: IR.RHC.REC.1400.038). The registered records of adult patients undergoing coronary artery bypass graft surgery at Shahid Rajaei Heart Hospital, Tehran, Iran, between April 2020 and April 2021 (about 500 patients) were examined in this study with 395 patients entered into the study based on the inclusion and exclusion criteria. The inclusion criterion included40-70-year-old patients who underwent coronary artery bypass graft surgery, and the exclusion criteria included emergency ward patients, patients with renal problems (acute or chronic renal failure), patients who were re-transferred to the operating room after surgery due to bleeding or tamponade, patients who needed to continue mechanical ventilation for more than 24 hours, and patients who needed to embed balloon pump and extracorporeal membrane oxygenation (ECMO). Renal dysfunction was considered according to the RIFLE criteria. Acute renal failure is defined as increased serum creatinine of ≥ 0.3 mg/dL compared to the baseline serum creatinine level, serum creatinine absolute level equal to 4 mg/dL, or increased serum creatinine higher than 3 times compared to its baseline level, and also decreased urinary output ≤ 0.3 mL/kg per hour for more than 12 hours or anuria for more than 12 hours.

 Patients were classified into 4 groups based on the difference between the maximum lactate levels measured during surgery and their baseline level. These four groups were as follows: Group 1: Lactate level ≤ 0.1, Group 2: Lactate level between 0.1 and 0.9, Group 3: Lactate level between 1 and 1.9, and Group 4: Lactate level ≥ 2. All preoperative, intraoperative, and postoperative(in the ICU) data were collected using a standard sheet and then statistically analyzed. Out of approximately 500 records, 395 patients were entered into the study.

 In this study, patients’ demographic information, history of comorbid diseases such as diabetes and blood pressure, creatinine and urea levels, blood sugar, hemoglobin, and hematocrit before and after surgery were recorded. The intraoperative and postoperative use of inotropes and the durations of surgery, cardiopulmonary bypass (CPB) pump, and aortic cross-clamping were also recorded. Moreover, the amounts of urine output during surgery and in the ICU were also extracted. Inpatient records, arterial blood analysis at the time of entering the operating room, after intubation and mechanical ventilation, after the patient’s putting on the cardiopulmonary bypass pump, and when rewarming and being separated from the cardiopulmonary bypass were considered and recorded. Also, after transferring the patient to the ICU, these arterial blood analysis samples were carried out every 1 hour in the first 4 hours and then every 2 hours, whose related information was also recorded. In this study, the patient’ s intubation duration in the ICU and the duration of hospitalization in the ICU and the hospital were also considered.

###  Statistical analysis

 All data were analyzed using SPSS software (version 26) at a significance level of 5%. Mean and standard deviation were used to describe quantitative variables, and frequency and percentage were used for qualitative variables. Repeated measures analysis of variance (ANOVA) was used to compare paired variables at repeated times. We used one-way ANOVA to compare preoperative quantitative variables between lactate change groups.

## Results

 In the current study, 395 patients were investigated and analyzed. The patients’ demographic information is provided in Table 1. In this study, 75.3% of patients were male and 24.3% were female (mean age = 57.98 years); 48.3% of patients had diabetes, and 60% had a history of high blood pressure; the mean surgery duration was 4.47 ± 0.62 hours, the mean cardiopulmonary bypass time was 68.63 ± 23.99 minutes, the mean aortic cross-clamping time duration was 37.49 ± 13.01 minutes, and the mean intubation duration in the ICU was 12.65 ± It is 4.97 hours. Moreover, the mean durations of hospitalization in the ICU and in the hospital were 3.57 ± 0.77 and 10.45 ± 2.44 days, respectively. The intraoperative arterial blood sample analysis indicated that the trend of changes in parameters such as lactate, hemoglobin, hematocrit, and blood sugar was ascending and statistically significant (*P *value = 0.001) compared to the baseline state (Table 2), although the trend of changes of these variables in the ICU was not significant. In addition, a descending trend in pH, bicarbonate level, and base excess was observed in the arterial blood sample during surgery, which was statistically significant (*P *value = 0.001) (Table 2). The trend of changes in urinary output at different times during pumping was first ascending and then descending, which was statistically significant (*P *value = 0.001), but the changes in the urine output level in the ICU were not statistically different. No significant relationship was observed in this study between blood urea nitrogen(BUN)and creatinine before and after surgery in the ICU and 24 hours postoperative in the ICU (Table 3); 100(25.3%) patients received intraoperative inotrope and 95(24.05%) received inotrope in the first 6 hours of hospitalization in the ICU; 173 (43.7%) patients received packed cell in the operating room, 251 (63.5%) received platelets in the operating room, and 140 (35.44%) received fresh frozen plasma (FFP). In the first 6 hours of hospitalization in the ICU, 99 (25.06%) patients received packed cells, 40 (10.12%) were injected with platelets, and 47 (11.8%) received FFP. In this study, three patients needed postoperative dialysis. The trend of lactate, blood sugar, hemoglobin, and hematocrit changes were generally compared between dialysis patients and non-dialysis patients, and the changes were statistically significant only in terms of the hematocrit variable between these two groups (*P* value = 0.04). The trend of lactate and hemoglobin changes was higher among dialysis patients. The mean BUN and creatinine levels before and after surgery in the ICU and 24 hours postoperative in the ICU were higher in dialysis patients than in non-dialysis patients, which was statistically significant (*P *value = 0.001).All cases undergoing dialysis (n = 3) had lactate levels less than 2.5 mmol/L upon entering the operating room, after intubation, during rewarming, and upon being separated from the cardiopulmonary bypass (not placed in the hyperlactatemia group), and at the time of cardiopulmonary bypass, only one of these dialysis patients was in the hyperlactatemia group, and none of the patients who were in the hyperlactatemia group at different times in the operating room (except one case) underwent dialysis. In Table 4, considering one of the references, the patients were classified into 4 groups based on the difference between the maximum lactate level measured during surgery and its base level. These four groups were as follows: Group 1: Lactate level ≤ 0.1, Group 2: Lactate level between 0.1 and 0.9, Group 3: Lactate level between 1 and 1.9, and Group 4: Lactate level ≥ 2. This classification demonstrated no significant relationship between BUN before and after surgery, 24 hours after surgery and intraoperative lactate changes. Furthermore, no significant relationship was found between creatinine before surgery and 24 hours after surgery and intraoperative lactate changes, but a significant relationship was found between postoperative creatinine and intraoperative lactate changes (*P* value = 0.05). Moreover, significant associations were observed between the cardiopulmonary bypass duration (*P* value = 0.02) and intraoperative inotrope infusion (*P* value = 0.03) and inotrope infusion during the first six hours in the ICU (*P* value = 0.049); receiving packed cell (*P* value = 0.006) and platelets during surgery (*P* value = 0.04) were found to have significant associations with intraoperative lactate changes. In the current research, the duration of hospitalization in the ICU and the hospital had no significant association with intraoperative lactate changes.

**Table 1:** Basic (demographic) characteristics of the population under investigation

**Variables**	
Age, mean (SD)	57.98 (7.71)
Gender, N (%) Male Female	299 (75.6) 96 (24.3)
Height, mean (SD)	167.95 (9.18)
Weight, mean (SD)	77.28 (13.11)
BMI, mean (SD)	27.24 (3.69)
Diabetes mellitus, N (%) Yes No	191 (48.3) 204 (51.6)
Hypertension, N (%) Yes No	237 (60) 158 (40)

SD: Standard deviation; N: Number; BMI: Body mass index

**Table 2:** Changes in intraoperative arterial blood sample variables

**Variables**	**Entrance** **Mean (SD)**	**After Intubation** **Mean (SD)**	**On Pump** ** Mean (SD)**	**Rewarming** **Mean (SD)**	**Pump Weaning** **Mean (SD)**	***P*** ** value**
Lactate	0.74 (0.35)	0.84 (0.43)	2.06 (10.41)	1.74 (0.76)	1.95 (1.75)	0.001
FBS	104.94 (33.02)	107.50 (35.29)	127.58 (41.49)	143.71 (42.89)	143.86 (43.53)	0.001
HB	14.10 (1.51)	12.84 (3.36)	9.95 (1.74)	10.11 (5.87)	10.25 (1.31)	0.001
HCT	45.52 (4.97)	41.22 (6.24)	32.12 (5.85)	31.66 (4.20)	33.24 (4.51)	0.001
PH	7.44 (0.02)	7.42 (0.05)	7.51 (0.91)	7.39 (0.05)	7.38 (0.06)	0.001
PO2	126.98 (107.32)	358.22 (125.17)	441.74 (125.34)	363.93 (133.12)	296.68 (127.62)	0.001
O2SAT	96.89 (1.69)	99.84 (0.61)	99.98 (0.13)	99.87 (0.42)	99.56 (0.85)	0.001
PCO2	39.82 (5.59)	38.24 (6.24)	38.92 (5.54)	36.96 (5.43)	36.18 (4.99)	0.001
HCO3	26.77 (2.65)	26.50 (9.82)	23.57 (2.63)	23.14 (2.71)	22.13 (2.91)	0.001
TCO2	27.53 (2.64)	26.17 (2.68)	24.79 (3.20)	24.01 (2.47)	22.96 (2.56)	0.001
BE	3.04 (2.16)	1.66 (2.72)	-0.05 (2.75)	-0.92 (2.67)	-1.78 (2.81)	0.001

FBS: Fasting blood sugar mg/dl; HB: Hemoglobin g/dl; HCT: Hematocrit %; PO2: Partial pressure of oxygen; O2SAT: Oxygen saturation; PCO2: Partial pressure of carbon dioxide; HCO3: Bicarbonate mg/l; TCO2: Total carbon dioxide; BE: Base excess, *P* value based on Independent Samples T-test. *P* < 0.05 statistically significant.

**Table 3:** Mean changes of urea and creatinine preoperative, postoperative in the intensive care unit, and 24 hours postoperative in the intensive care unit

	**Before Surgery** **Mean±SD**	**After surgery** **Mean±SD**	**24 Hours after Surgery** **Mean±SD**	***P*** ** value**
BUN	15.53 ± 5.13	15.28 ± 4.94	15.35 ± 5.30	0.51
CR	1.10 ± 0.83	1.08 ± 1.06	1.11 ± 1.22	0.87

BUN: Blood urea nitrogen mg/dl; CR: Creatinine mg/dl, *P* value based on Independent Samples T-test. *P* < 0.05 statistically significant.

**Table 4:** The correlations of intraoperative and postoperative patient outcomes with lactate changes

**Variable **	**Total (n=395)**	**Intraoperative Change in Lactate Level, mmoL**				***P*** ** value**
		**≤0.1, (n=6)**	**0.1 to 0.9 (n=119)**	**1 to 1.9 (n=192)**	**≥2 (n=78)**	
CPB time(minute)	68.63 ± 23.99	54.16 ± 9.32	66.63 ± 19.26	67.58 ± 24.86	75.38 ± 27.39	0.02
Aortic cross-clamp time (minute)	37.49 ± 13.01	33.16 ± 7.54	37.47 ± 10.88	36.43 ± 13.41	40.42 ± 14.95	0.11
Surgery time(hour)	4.47 ± 0.62	4.38 ± 0.48	4.48 ± 0.59	4.44 ± 0.63	4.53 ± 0.64	0.75
ICU hospitalization (day)	3.57 ± 0.77	3.66 ± 0.81	3.47 ± 0.47	3.59 ± 0.86	3.68 ± 0.69	0.28
Total hospitalization (day)	10.54 ± 2.44	10 ± 1.67	10.61 ± 2.41	10.41 ± 2.56	10.79 ± 2.23	0.63
Postoperative ventilation time (hour)	12.65 ± 4.97	12.33 ± 2.94	12.51 ± 6.31	12.97 ± 4.65	12.24 ± 3.34	0.74
Intraoperative inotrope	Yes = 100	0	27(22.6)	44(23.03)	29(37.66)	0.03
	No = 295	8(100)	92(77.31)	147(76.96)	48(62.33)	
First 6 hours ICU inotrope	Yes = 95	0	27 (23.27)	41 (21.24)	27 (36)	0.049
	No = 300	11 (100)	89 (76.7)	152 (78.7)	48 (64)	
Intraoperative packed cell	Yes = 173	2 (28.5)	59 (50)	68 (35.4)	44 (56.4)	0.006
	No = 222	5 (71.4)	59 (50)	124 (64.6)	34 (43.6)	
Intraoperative platelets	Yes = 251	4 (57.1)	63 (53.4)	132 (68.8)	52 (66.7)	0.04
	No = 144	3 (42.8)	55 (46.6)	60 (31.3)	26 (33.3)	
Intraoperative FFP	Yes = 140	3 (42.8)	31 (26.3)	73 (38)	33 (42.3)	0.07
	No = 255	4 (57.1)	87 (73.7)	119 (62)	45 (57.7)	
First 6 hours ICU packed cell	Yes = 99	2 (18.1)	27 (23.1)	51 (26.7)	19 (25)	0.81
	No = 296	9 (81.8)	90 (76.9)	140 (73.3)	57 (75)	
First 6 hours of ICU platelets	Yes = 40	1 (9.09)	12 (10.3)	14 (7.3)	13 (17.1)	0.08
	No = 355	10 (90.9)	105 (89.7)	177 (92.7)	63 (82.9)	
First 6 hours ICU FFP	Yes = 47	0	17 (14.5)	17 (8.9)	13 (17.1)	0.17
	No = 343	11 (100)	100 (85.5)	174 (91.1)	63 (82.9)	
BUN preoperative	15.53 ± 5.13	17.50 ± 4.18	15.39 ± 5.39	15.37 ± 5.17	15.57 ± 4.73	0.81
BUN postoperative	15.29 ± 4.94	15.83 ± 3.12	15.51 ± 5.25	14.81 ± 4.38	16.08 ± 5.73	0.25
BUN 24 hours postoperative	15.34 ± 5.29	15 ± 3.52	15.62 ± 5.43	14.85 ± 4.87	16.13 ± 6.11	0.29
CR preoperative	1.10 ± 0.83	0.88 ± 0.21	1.07 ± 0.24	1.13 ± 1.17	1.07 ± 0.22	0.82
CR postoperative	1.08 ± 1.06	0.81 ± 0.19	1.01 ± 0.25	1.01 ± 0.24	1.38 ± 2.33	0.05
CR 24 hours postoperative	1.11 ± 1.22	0.80 ± 0.2	1.05 ± 0.31	1.08 ± 0.71	1.30 ± 2.51	0.46

CPB: Cardiopulmonary bypass minute; ICU: Intensive care unit; FFP: Fresh frozen plasma; BUN: Blood urea nitrogen mg/dl; CR: Creatinine mg/dl, *P* value based on Chi-square test and Independent Samples T-test (variables containing yes and no subgroups analyzed with Chi-square test and variables with mean (SD) were analyzed by Independent Samples T-test). *P* < 0.05 statistically significant.

## Discussion

 According to the findings of the present research, blood lactate level alone is not a reliable diagnostic and predictive marker for renal dysfunction after cardiac surgery in adults. In this study, no significant relationship was observed between intraoperative lactate changes and preoperative and postoperative BUN and 24 hours post-operative and also between preoperative creatinine and 24 hours postoperative, although postoperative lactate and creatinine changes were found to be significantly correlated. Moreover, three patients needed postoperative dialysis, and only one of them who needed dialysis at the time of cardiopulmonary bypass was in the group with lactate over 2.5, indicating that intraoperative lactate changes are not an appropriate marker to evaluate postoperative renal function. Although some studies have shown that blood lactate level is a biomarker for early diagnosis of acute renal failure, and many studies have been conducted on the relationship between lactate levels and mortality rates after cardiac surgery, Basaranet al (2006) have indicated that in patients undergoing cardiac surgery, postoperative lactate level is associated with higher mortality rate, longer stay in the ICU, longer hours of mechanical ventilation, and more need for inotrope medicines and renal alternative treatments.^9^ Our study indicated a significant relationship between intraoperative lactate changes and inotrope infusion during surgery and six hours after surgery in the ICU, but these intraoperative lactate changes had no significant association with the duration of hospitalization in the ICU and the hospital. Ina survey conducted by Argwal et al (2012), it was suggested that the investigation of dynamic parameters could identify patients in need of more precise interventions.^10^ In this study, it was also demonstrated that during surgery, the trend of changes in parameters such as lactate, hemoglobin, hematocrit, and blood sugar was ascending, which was statistically significant. The relationship between postoperative outcomes and intraoperative lactate changes was assessed in the current research, and it is suggested that more complete studies should be conducted for other parameters. Zhang Z et al’s study (2015) investigated serum lactate levels and indicated the existence of a relationship between the blood level of this biomarker and patients with postoperative acute renal injury after cardiac surgery; they also showed that a significant increase in serum lactate could stem from the lack of blood in renal tissues during cardiac revascularization,^6^ while are inconsistent with our findings; in addition, there is no significant relationship was observed between the patients in the hyperlactatemia group during surgery and postoperative renal function, demonstrating that numerous variables are required to be assessed in this regard and lactate alone cannot be a reliable marker. Ina retrospective study by Naik R et al (2016) to identify factors causing high lactate levels in patients undergoing cardiopulmonary bypass surgery, after evaluating the association between high blood lactate levels and mortality rates after surgery, it was found that hyperlactatemia was significantly related to post-operative complications and its diagnosis should be taken into account during surgery.^11^ Paying attention to hyperlactatemia is important in our research, but it is not decisive as an independent marker. Of course, in this study, the hyperlactatemia level was defined as a blood lactate level ≥ 4 mmol/L, whereas in our study, the hyper lactatemia level was defined as ≥ 2.5 mmol/L. In a study by Minton J et al (2017), multiple factors were found to contribute to the production and metabolism of lactate during the postoperative phase, not stemming from hypoxic and non-hypoxic mechanisms,^7^ which is consistent with our study because only 3 out of 395 patients underwent dialysis, their lactate levels were less than 2.5 mmol/L when entering the operating room, after intubation, during rewarming, and upon being separated from cardiopulmonary bypass (not placed in the hyperlactatemia group) and only one patient was in the hyperlactatemia group during cardiopulmonary bypass, and of all patients in this study, the same one patient underwent dialysis; the lactate (hyperlactatemia) level in the postoperative phase and during the stay in the ICU may provide a more reliable predictor. Haanschoten M.C. et al’s (2017) study revealed that the measurement of intraoperative lactate level alone was not a useful indicator for prognosis.^12^ Duval B et al (2019)compared the relationship between the 30-day total lactate level in the ICU and the mortality rate in a cardiac surgery hospital and showed the association of high lactate levels with poor outcomes in adults undergoing cardiac surgery.^13^ However, this research has indicated that high lactate levels alone are not decisive, and other factors and parameters should also be taken into consideration. Similarly, lactate levels in the first six hours of stay in the ICU are not enough, and lactate levels should also be taken into account in the following days of ICU hospitalization. Govender P et al (2020) conducted a retrospective study to determine the importance of the increased lactate levels independent of the absolute lactate level during induction of anesthesia as a dynamic parameter to examine complications during patient stay in the ICU, renal failure, use of inotrope, and adult mortality after cardiac surgery. In this study, lactate was assessed in five defined groups, and it was indicated that patients in Group 5 had four times higher mortality rates (7.7%) than those in Group 1 (lactate level increased compared to Group 1) and the ICU hospitalization duration was longer in this group of patients. They also suffered from the most acute cases of postoperative renal failure (19.2%).In this investigation, the increased intra operative lactate levels predicted mortality rate and long ICU hospitalization and renal failure that required alternative treatments, which were statistically significant and concluded that the increased intraoperative blood lactate levels independent of its amount during anesthesia induction was a beneficial dynamic parameter to detect patients at risk of postoperative mortality and could be effective in preventing poor outcomes.^14^ Furthermore, there was a significant association in our study between the duration of cardiopulmonary bypass and inotrope infusion during surgery and inotrope infusion during the first six hours in the ICU; also, receiving intraoperative packed cell and platelet and postoperative creatinine were significantly related to intraoperative lactate changes. Moreover, in this study, the duration of hospitalization in the ICU and the hospital was not found to be significantly correlated with intraoperative lactate changes. Duval B. et al’s (2019) study to compare the relationship between the 30-day total lactate levels in the ICU and mortality in a cardiac surgery hospital demonstrated that high lactate levels were associated with poor outcomes in adults undergoing cardiac surgery.^13^

## Conclusion

 The present study demonstrated that patients’ intraoperative blood lactate levels had no obvious significant associations with postoperative renal function and renal function at 24 hours postoperative. These findings also indicate that changes in intraoperative blood lactate levels alone are not a predictor for renal dysfunction and long stays in the ICU and the hospital in adults undergoing cardiac surgery. Thus, blood lactate level independently seems to be an unreliable marker for predicting renal dysfunction and long stays in the ICU and the hospital in adults undergoing cardiac surgery.

## Competing Interests

 There was no conflict of interest.

## Ethical Approval

 The study was approved by the Ethics Committee of in Rajaie Heart Center (code: IR.RHC.REC.1400.038).
